# P363: Qualitative and quantitative evaluation of medical waste products in Côte d'Ivoire

**DOI:** 10.1186/2047-2994-2-S1-P363

**Published:** 2013-06-20

**Authors:** MJ Bitty, OP Kamelan, YB Coha, JD Seka, M Samba

**Affiliations:** 1 Dircetion of Public Hygiene, Ministry of Health and fight against Aids, Abidjan, Côte d'Ivoire

## Introduction

Medical waste is now a real public health problem because of their negative impacts on health and the environment. Since the studies in 2002, Côte d'Ivoire has not yet conducted a qualitative and quantitative assessment of medical waste generation. This makes it difficult to put in place a coherent strategy for waste management, planning and design of some management structures. This evaluation of medical waste reflects a desire to update the data for better decision making.

## Objectives

The overall objective is to evaluate qualitatively and quantitatively the medical waste in Côte d'Ivoire.

## Specific Objectives

Identify medical waste according to a typology; Know the amount of each type of medical waste; Make recommendations.

## Methods

The structures are the structures of the health system in the private sector and the public. The selection of structures was carried out through a survey at 2 levels. All survey sites were monitored at least 3 times during the investigation. The collection includes two steps: sorting and weighing of waste. Each site was equipped with bins 27 and 80 liters of blue garbage bags (infectious medical waste and medical waste non-infectious), black garbage bags (waste comparable to domestic waste), safety box for sharp objects, spikes and / or sharp and a precision electronic balance (2 grams).

## Results

The daily domestic production of medical waste in the public sector is estimated at 11,617,739 g or 11.62 tons. Domestic production in the private sector is estimated at 1,476,997 g per day, or 1.48 per ton. Infectious and hazardous wastes represent 59.39% of the medical waste. Côte d'Ivoire produces daily 13.1 tonnes of medical waste is estimated to be 4 781.5 tonnes annually in all sectors.

## Conclusion

Nearly 70% of medical waste is infectious and dangerous. The results are for the public sector, 809 g / bed / day against 452 g / bed / day for the private sector. The national average is estimated at 630g/bed / day.

## Disclosure of interest

None declared

